# TRPV1: A Potential Drug Target for Treating Various Diseases

**DOI:** 10.3390/cells3020517

**Published:** 2014-05-23

**Authors:** Rafael Brito, Sandeep Sheth, Debashree Mukherjea, Leonard P. Rybak, Vickram Ramkumar

**Affiliations:** 1 Department of Pharmacology and Neuroscience, Southern Illinois University School of Medicine, Springfield, IL 62702, USA; E-Mails: rafaelbs2002@yahoo.com.br (R.B.); ssheth@siumed.edu (S.S.); lrybak@siumed.edu (L.P.R.); 2 Department of Neurobiology and Program of Neurosciences, Institute of Biology, Fluminense Federal University, Niteroi 24020141, RJ, Brazil; 3 Department of Surgery (Otoloryngalogy), Southern Illinois University School of Medicine, Springfield, IL 62702, USA; E-Mail: dmukherjea@siumed.edu

**Keywords:** TRPV1, peripheral neuropathy, ototoxicity, diabetes, cystitis, obesity

## Abstract

Transient receptor potential vanilloid 1 (TRPV1) is an ion channel present on sensory neurons which is activated by heat, protons, capsaicin and a variety of endogenous lipids termed endovanilloids. As such, TRPV1 serves as a multimodal sensor of noxious stimuli which could trigger counteractive measures to avoid pain and injury. Activation of TRPV1 has been linked to chronic inflammatory pain conditions and peripheral neuropathy, as observed in diabetes. Expression of TRPV1 is also observed in non-neuronal sites such as the epithelium of bladder and lungs and in hair cells of the cochlea. At these sites, activation of TRPV1 has been implicated in the pathophysiology of diseases such as cystitis, asthma and hearing loss. Therefore, drugs which could modulate TRPV1 channel activity could be useful for the treatment of conditions ranging from chronic pain to hearing loss. This review describes the roles of TRPV1 in the normal physiology and pathophysiology of selected organs of the body and highlights how drugs targeting this channel could be important clinically.

## 1. Introduction

TRPV1 channels serve primarily as heat sensors, which are activated by temperatures >43 °C. These channels are present on sensory neurons, such as dorsal root ganglia and trigeminal neurons, where they are localized primarily in small diameter neurons and unmyelinated C fibers [1]. Amino acid sequence data predict a channel with six trans-membrane spanning regions and a pore located in a hydrophobic stretch between transmembrane segments 5 and 6. The pore shows a greater selectivity for Ca^2+^ over Na^+^ of 9.6:1 [1]. TRPV1 is also activated by capsaicin, an active ingredient in hot chili peppers [1,2]. Heat and capsaicin increase Ca^2+^ currents in cells expressing TRPV1. Protons (pH < 5.9) can directly activate TRPV1 channels and further enhance the sensitivity of these channels to capsaicin and heat [1]. Protons-induced activation could be relevant in tissue ischemia or inflammation. Thus, TRPV1 serves as an integrator of physical and chemical stimuli produced from the injury site or from external sources [1,2].

Chronic capsaicin administration desensitizes TRPV1 and renders the neurons less sensitive to noxious (painful) stimuli. This action requires the presence of extracellular Ca^2+^ and activation of Ca^2+^-calmodulin dependent protein kinase which promote channel phosphorylation [1,2]. This property of capsaicin has been employed for the treatment of pain associated with disease conditions such as diabetic peripheral neuropathy and arthritis [1,2].

## 2. Endogenous TRPV1 Agonists

These agonists, also referred to as endovanilloids, are expressed predominantly in the primary sensory neurons [3] and also different regions of the brain [4,5]. Endovanilloids are synthesized in the cells and released in an activity-dependent manner in adequate amounts to evoke TRPV1-mediated responses [6]. The endovanilloid signaling is terminated within a short period of time, which allows for strict control of its action.

**Figure 1:**
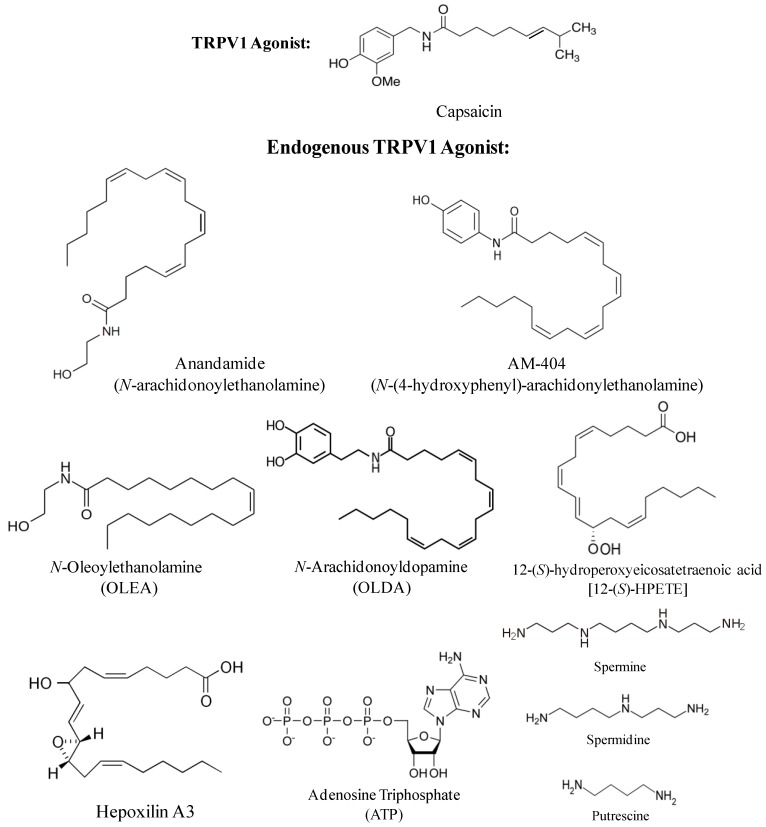
Chemical structures of natural and endogenous transient receptor potential vanilloid 1 (TRPV1) receptor agonists.

Various endogenous lipids from the fatty acid pool have been identified as TRPV1 activators. Anandamide (*N*-arachidonoyl ethanolamine) (Figure 1), the endogenous ligand for the cannabinoid receptors, was reported to activate TRPV1 by binding to the same site as capsaicin [7]. However, its potency was 5–10 fold lower than that of capsaicin [8]. Endogenously produced anandamide causes TRPV1-dependent ileitis in the inflamed ileum of rats treated with *Clostridium difficile* toxin A [9]. AM-404 [*N*-(4-hydroxyphenyl)-arachidonoyl-ethanolamine] (Figure 1), an anandamide reuptake inhibitor and TRPV1 agonist, has been shown to attenuate motor disturbances by restoring GABA and dopamine transmission in an animal model for Huntington’s disease [10,11]. *N*-acyl ethanolamines (NAEs), like anandamide, have been studied as activators of TRPV1. One such NAE, *N*-oleoylethanolamine (OLEA) (Figure 1), evokes TRPV1 currents in cells previously sensitized with protein kinase C [12]. Intraperitoneal administration of OLEA induces visceral pain behavior in wild type, but not in TRPV1-null mice [13].

*N*-arachidonoyldopamine (NADA) (Figure 1), an endocannabinoid present in CNS, is a potent full agonist of TRPV1, with 5–10 fold higher potency than anandamide and equi-potent to capsaicin in functional assays [14]. Intradermal injection of NADA into the hind paw of mice results in thermal hyperalgesia [15]. NADA also constricts isolated bronchi and urinary bladder preparations from the guinea pig in a TRPV1-dependent manner [16]. A bioactive analogue of NADA, *N*-oleoyl dopamine (OLDA) (Figure 1), evokes increase in intracellular Ca^2+^ in TRPV1 expressing HEK293 cells. Similar to NADA, subcutaneous injection of OLDA produces thermal hyperalgesia [17].

Various lipooxygenase products of arachidonic acid, such as 5-(*S*), 8-(*S*), 12-(*S*) and 15-(*S*)-hydroperoxyeicosatetraenoic acids (HPETEs), are derived from the insertion of oxygen into the double bonds of the aliphatic chain of arachidonic acid. Since this reaction is catalyzed by lipoxygenases, these derivatives are called lipooxygenase products [18]. Among different HPETEs, 12-(*S*) HPETE (Figure 1) showed the highest potency to evoke capsazepine (a TRPV1 antagonist)-sensitive currents in DRG neurons and in HEK cells expressing TRPV1 [19]. It was reported that 12-(*S*) HPETE is produced in sensory neurons upon stimulation of sensory nerve endings by the inflammatory mediator bradykinin. In this study, bradykinin was shown to activate cAMP-dependent phospholipase 2 (PLA2) leading to the release of arachidonic acid, which is then metabolized by 12-lipoxygenase to produce 12-(*S*) HPETE. This pathway has been linked to thermal hyperalgesia induced by bradykinin [20]. In addition, 12-(*S*) HPETE can undergo other enzymatic reactions that lead to the formation of hepoxilins A3 (HXA3) and B3 (HXB3) (Figure 1). Intrathecal injections of these hepoxilins induce tactile allodynia via activation of TRPV1 (and TRPA1) in rats [21].

In addition to the endogenous lipids, other endogenous chemicals, including ATP, ammonia and polyamines (such as spermine, spermidine, and putrescine), as well as protons (observed during inflammation) can activate TRPV1 (for review see [22,23]) (Figure 1). ATP was identified as a TRPV1-sensitizing molecule that directly binds to TRPV1 in a region between ankyrin repeats 1–3 [24]. Moreover, by activating purinergic P_2_ receptors on sensory neurons, ATP stimulates phospholipase C (PLC) which produces diacylglycerol (DAG), an activator of PKC. Activators of PKC have been shown to sensitize TRPV1 channels to endogenous and exogenous agonists and reduce the temperature threshold for channel activation [25,26]. Acidic conditions normally occur during hypoxia, ischemia and inflammation [27]. Protons decrease the temperature threshold for TRPV1 activation such that even moderately acidic conditions (pH≤5.9) can activate the channel at room temperature. TRPV1 can also be activated by basic pH. Ammonia (NH_3_), an irritant and weak base, activates TRPV1 (and TRPA1) in sensory neurons through a mechanism that involves a cytoplasmic histidine residue [28]. Polyamines are capable of modulating inflammation and nociception by regulating the activity of TRPV1 channel. Extracellular spermine, spermidine, and putrescine, by virtue of their cationic charge, directly activate TRPV1 in a charge-dependent manner, both in heterologous expression systems and in sensory neurons. The threshold for activation by spermine is rather high (~500 μM at room temperature), but spermine can enhance capsaicin evoked currents with an effective concentration for 50% activation (EC_50_) value of approximately 5 μM [29].

## 3. Functions of TRPV1 Channel

### 3.1. Role of TRPV1 in Thermal Sensation

One of the remarkable features of living organisms is their ability to perceive and react to environmental conditions around them. The skin of animals has an ability to identify thermal profiles ranging from cold to extreme heat. Within this range, temperatures above 43 °C and below 15 °C evoke painful thermal sensations by activating C type nerve fibers [30,31]. Pioneering studies showed that primary afferent neurons express heat-sensitive ion channels [32,33] which were later cloned and identified as TRPV1 receptor [1,3]. The mRNA encoding TRPV1 receptor was expressed in small diameter sensory neurons within the dorsal root ganglion and trigeminal sensory ganglia, consistent with the idea that C and A-δ fibers are involved in nociception [3,34]. Immunolabeling for TRPV1 is observed in non-myelinated, low conductance nerve fibers which express neuropeptides such as substance P and neurokinin A [34,35]. TRPV1 was activated at temperatures >43 °C which are responsible for inducing pain *in vivo* [3,34]. TRPV1 knockout mice exhibit a reduced response to noxious stimuli induced by capsaicin, heat and protons [36,37,38]. Neurons of the dorsal root ganglion obtained from TRPV1 null mice showed greatly reduced sensitivity to heat, but a small number of neurons maintained their response when stimulated with heat temperatures above 50 °C, indicating that other receptors participate in pain perception in this temperature range [36]. The membrane potential and excitability of magnocellular neurosecretory cells of mice maintained at physiological temperature is reduced by genetic deletion or pharmacological blockade of the TRPV1 receptors. Moreover, the spontaneous electrical activity of these cells is abolished by the same experimental profile, indicating that TRPV1 plays a physiological role in the thermal sensation [39]. Human corneal epithelial cells also express functional TRPV1 receptors which are responsive to capsaicin and heat. Treatment with selective agonist 2-aminoethoxydiphenyl borate (2-APB), or exposure to temperatures above 40 °C, led to a transient increase in intracellular Ca^2+^ concentration, which was blocked by pretreatment with selective TRPV1 antagonists [40].

Inflammatory pain is produced following tissue damage and/or development of inflammation. It is characterized by hypersensitivity of the site of damage and nearby tissues to noxious stimuli. One of the mechanisms responsible for this aspect of inflammation is the modulation of ion channels, such as TRPV1, which become sensitized by mediators in the inflammatory micro-environment [41,42,43]. These mediators include prostaglandins, bradykinin, serotonin, ATP and adenosine [27]. Among these molecules, ATP, bradykinin, and prostaglandins sensitize TRPV1 through activating Ca^2+^ mobilizing receptors and PKC. These mediators reduce the threshold temperature and proton concentrations required to activate TRPV1 [26,44,45,46,47,48]. Studies using TRPV1 knockout mice and knockout of receptors for inflammatory mediators demonstrate essential roles for TRPV1 and inflammatory mediators for producing inflammatory pain [45,46,47]. The cytosolic domain of TRPV1 contains serine residues which could be phosphorylated by PKC [49,50]. Recently, it was shown in the neurons of the dorsal root ganglion and transfected cells that cyclin-dependent kinase 5 (Cdk5) controls trafficking of TRPV1 to the cell membrane through kinesin-3 family (KIF) 13B protein. Interestingly, activation of Cdk5 by injection of Freund’s adjuvant increases the trafficking of TRPV1 to the cell membrane, which likely contributes to development and maintenance of hyperalgesia [51]. The activity of TRPV1 could also be enhanced when it is associated with another receptor. In this sense, Cheng *et al.* [52] showed that TRPV1/TRPV3 heterodimers exhibit increased temperature sensitivity, lower threshold for activation and sensitization by heat as compared to the monomeric receptors. The microenvironment created by tissue damage and inflammation is rich in protons produced by inflammatory cells. By activating TRPV1 in these inflamed areas, protons can also contribute to pain sensations [53,54]. Thus, acidosis can enhance pain. Previous studies have shown that acidosis sensitizes visceral and cutaneous nociceptors, in large parts through activation of TRPV1 [55,56,57]. In extreme acidosis, protons can directly activate TRPV1. However, in mild acidic conditions, protons act as allosteric modulators of TRPV1 and greatly increase their sensitivity to heat, capsaicin and inflammatory agents. This phenomenon is typical of disorders characterized by increased tissue acidosis [53].

In addition to the sensitization of TRPV1 by inflammatory mediators and protons, activation of TRPV1 can enhance the release of inflammatory molecules associated with pain transmission, such as substance P, calcitonin gene-related peptide (CGRP) and bradykinin, which can contribute to peripheral sensitization of TRPV1 [58]. Moreover, TRPV1 expression is up-regulated flowing inflammation and nerve damage which can further enhance the responses mediated by these receptors [59,60].

### 3.2. Role of TRPV1 in Diabetes and Obesity

Type 1 diabetes has an autoimmune basis involving T cell-targeted destruction of pancreatic islet β cells. In this disease, autoreactive T cells target antigens on islet β cells or neurons, initiating a local inflammatory response and destruction of these β cells. These antigens include glutamic acid decarboxylase [61,62], myelin based proteins [63], glial fibrillary acid proteins and S100β [64]. TRPV1 is expressed on nerves innervating the islet β cells where they appear to modulate T cell function. An early study demonstrated that administration of capsaicin to neonatal mice was able to destroy these TRPV1 expressing neurons [65]. Interestingly, this treatment protected these mice from autoimmune diabetes [66]. It is believed that TRPV1 expressing neurons could induce the type 1 diabetic phenotype by modulating the proliferation and activity of T cell in the microenvironment of the islet β cells through the release of substance P. Pancreatic islet cells also include resident dendritic cells which express TRPV1 receptors [67]. Activation of these receptors by capsaicin or endovanilloids could activate dendritic cell function which includes antigen presentation to CD4^+^ T cells and chemotaxis. Since TRPV1 neurons play a critical role in modulating inflammation at the level of the pancreatic β cells, it could serve as a useful target for controlling inflammation and reducing diabetic symptoms. This suggests the clinical utility of TRPV1 antagonists or agonist-induced desensitization of TRPV1 to treat type 1 diabetes. The expression of TRPV1 in dendritic cells has been corroborated by some studies [67,68,69] but not others [70]. The idea that TRPV1 is an initiating factor mediating type 1 diabetes is challenged by the finding that TRPV1 isoform expressed in the non-obese diabetic (NOD) mouse possesses two mutations which render the channel less active and thereby should be less able to regulate the inflammatory process [66]. However, this discrepancy has been explained as faulty control mechanism between pancreatic islet β cells and the innervating TRPV1 expressing neurons. TRPV1 receptors are also expressed on islet β cells where they control the release of insulin [71]. Since insulin could sensitize TRPV1 receptors [72], it could positively regulate TRPV1 activity and increase the release of CGRP.

Diabetic peripheral neuropathy is a complication associated with diabetes mellitus, resulting from damage of loss of peripheral nerve terminals innervating the extremities. Affected patients normally complain of pain, tingling, and loss of feeling in the extremities. Several studies have implicated TRPV1 in the development of diabetic peripheral neuropathy in animal models of type 1 diabetes [73,74] and in humans [75]. Hong and Wiley [73] showed upregulation of TRPV1 protein and channel activity in a streptozotocin (STZ)-induced diabetic rats in large myelinated A fibers, while the normally expressing C fibers showed reduced expression. In another study, Pabbidi *et al.* [74] demonstrated a direct correlation between TRPV1 expression in dorsal root ganglion cells and thermal sensitivity. These investigators showed that STZ-induced diabetic mice developed an early hyperalgesic response, followed by a later phase of hypoalgesia. A similar temperature sensitivity profile was demonstrated in a double transgenic model of diabetes [74]. Interestingly, in both of these models, increased TRPV1 levels in dorsal root ganglion cells were observed in the hyperalgesic phase, while reduced levels of this protein was obtained during the hypoalgesic phase. A likely explanation for these findings is that the level of TRPV1 dictates the thermal sensitivity of diabetic animals. Similar changes in TRPV1 levels in the skin were observed in humans with diabetic neuropathy [75,76].

Type 2 diabetes is believed to be associated with inflammation, as evidenced by high levels of C-reactive proteins (CRP) in patients [77,78]. Antidiabetic drugs, such as the peroxisome proliferator activated receptor γ (PPARγ) agonist pioglitazone, are able to lower blood glucose along with CRP [79]. However, it is yet unclear whether a link exists between PPARγ agonists, CRP and TRPV1. TRPV1 has also been implicated in insulin resistance and obesity, characteristic of type 2 diabetes. It is believed that localized inflammation in the pancreas leads to increase in the activity of TRPV1 associated with aging, which contributes to increasing levels of CGRP [80]. CGRP is known to promote insulin resistance and obesity [81] by decreasing insulin release from β cells [82]. Treatment of Zucker rats with capsaicin or resiniferatoxin (RTX) (to desensitize TRPV1 expressing neurons) reduced fasting plasma insulin and improved glucose tolerance [83]. These data suggest that targeting TRPV1 for inhibition could be a novel method for treating diabetes and insulin resistance.

Various pieces of data support the contention that consumption of capsaicin or endovanilloids helps to control food intake and obesity. For example, volunteers consuming capsaicin capsules showed increased satiety and increased energy expenditure [84]. A capsaicin containing preparation was also shown to aid in weight maintenance in obese individuals [85]. The administration of the endovanilloid N-oleoylethanolamide was shown to reduce food intake in wild type mice, but not in TRPV1 knockout mice, implicating TRPV1 in the control of food intake [13]. Overall, these data support a role of TRPV1 in the control of diabetes, insulin resistance and obesity.

### 3.3. TRPV1 in Ototoxicity of Cisplatin and Aminoglycoside Antibiotics

Platinum containing drugs have been successfully used in the treatment of various solid tumors, including tumors of the head and neck. One such drug, cisplatin, is an important component of chemotherapeutic regimens for treating these tumors. This drug produces ototoxicity, in part, through the generation of reactive oxygen species (ROS) [86]. One target of ROS includes the organ of Corti [87], where it destroys outer hair cells [88]. These cells play an important role in hearing. Current strategies to prevent hearing loss involve the use of antioxidants [87]. However, concomitant antioxidant use could interfere with the anticancer efficacy of cisplatin and limit its efficacy in chemotherapy. As such, other treatment targets have been sought after.

One such target is TRPV1, which is expressed in the organ of Corti and spiral ganglion cells [86]. Zheng *et al.* [89] showed that capsaicin affected several parameters of auditory function, such as increasing the threshold of auditory nerve compound action potential (CAP) and reducing the magnitude of cochlear microphonics and electrically evoked otoacoustic emissions. Capsaicin was also shown to produce a transient increase in cochlear blood flow [89], presumably by activating TRPV1 containing neurons innervating the spiral modiolar artery and arterioles and the stria vascularis [90]. These responses were inhibited by the TRPV1 antagonist capsazepine, implicating TRPV1 in mediating these actions. However, these investigators subsequently demonstrated inhibitory effects of suprathreshold concentrations of capsaicin (300 µM) on outer hair cells potassium channels (I_k_ and I_k,n_) [91]. A similar study by Zhou *et al.* [92] show increases in spiral ganglion activity following intracochlear perfusion of capsaicin.

An interesting observation is that TRPV1 and transduction channels in hair cells of the organ of Corti could gate the entry of different molecules into these cells [93]. These investigators observed rapid entry of FM1-43 styryl dye into the hair cells through stereocilia bundles on the apical surfaces of the cells [93]. Such a mechanism might allow the entry of a number of xenobiotics (such as cisplatin and aminoglycosides) into hair cells and sensory neurons. Similar findings were reported for the entry of gentamicin in Madin-Darby canine kidney (MDCK) cells [94] and in organ of Corti explant cultures [95].

In a recent study [96], we showed that TRPV1 is a target of ROS generated by cisplatin. ROS promote activation and induction of TRPV1 and the NOX3 isoform of NADPH oxidase (a major source of ROS generation in the cochlea) in the rat organ of Corti and spiral ganglion cells. Generation of ROS via NOX3 was shown to be crucial to the activation and induction of TRPV1. Subsequent studies demonstrated that TRPV1 serves as an integrator of “noxious” stimuli to activation of the inflammatory cascade in the cochlea [97]. This process involved coupling of TRPV1 to NOX3 and signal transducer and activator of transcription 1 (STAT1) [97]. Similar studies have shown induction in TRPV1 in the spiral and vestibular ganglia of mice following administration of kanamycin for 14 d [98]. Mice challenged by a single intratympanic injection of gentamicin were found to have increased intensity of TRPV1 immunoreactivity in cochlear hair cells, spiral ganglion cells, vestibular sensory cells and vestibular ganglion cells two weeks after injection [99].

It is well established that outer hair cells in the basal turn of the cochlea are more susceptible to damage than are the cells in the apical regions. Organ of Corti explants from neonatal rat cochlea were treated with gentamicin for 24 h. The greatest damage was demonstrated in the basal turn of the cochlea. Red fluorescence was observed in the basal turn of inner and outer hair cells treated with Texas Red labelled gentamicin (GTTR) [95]. Explants treated with gadolinium or ruthenium red blocked the uptake of GTTR in hair cells in a dose-dependent manner. Gadolinium blocks Ca^2+^-permeant, mechanosensitive cation channels and ruthenium red, a noncompetitive antagonist of TRPV1, is also a blocker of numerous cation channels [95]. Systemic injection of GTTR in neonatal rats resulted in accumulation of fluorescent label in hair cells but also in other cells in the cochlea. The authors concluded that hair cell susceptibility to damage by aminoglycosides may depend on uptake of these drugs, and that the uptake was mediated in part by TRPV1 proteins [95].

In a rat model of salicylate-induced tinnitus, induction of TRPV1 in the cochlea was observed 2 h following intraperitoneal drug administration [100]. Increases in TRPV1 levels in the cochlea of rats were also observed 24 h and 2 weeks following noise exposure [101]. These authors suggested that the increases in TRPV1 could be one mechanism underlying the development of tinnitus.

The importance of TRPV1 in mediating cisplatin ototoxicity was shown by siRNA knockdown studies. In these studies, it was shown that round window administration of TRPV1 siRNA decreased cisplatin-induced damage to outer hair cells in the organ of Corti, as evidenced from scanning electron microscopy. Round window application of TRPV1 siRNA prior to cisplatin administration significantly reduced the percentage of hair cell loss observed. Knockdown of TRPV1 also reduced cisplatin-induced hearing loss, as evidenced by preservation of auditory brainstem responses [96].

Recent studies have implicated TRPV1 in mediating inflammation in the cochlea through activation of STAT1 via a ROS-dependent pathway. Activation of TRPV1 stimulates NOX3 activity, the generation of ROS and subsequent phosphorylation of Ser^727^ p-STAT1 by mitogen activated protein kinase (MAPK), and also increases expression of inflammatory mediators [97,102]. Overall, these studies suggest that inhibition of TRPV1 or its downstream effector in the cochlea could provide protection against hearing loss.

### 3.4. TRPV1 in the Bladder

Sensory neurons innervating the urinary bladder and urethra play important roles in innate reflexes involving the storage of urine and urination. Dysfunctions of these sensory systems are probably related to disorders, such as urinary incontinence. The upper and lower urinary tracts of human and other species are richly innervated by capsaicin-sensitive neurons [103,104] which are primarily unmyelinated C nerve fibers [105]. These sensory neurons regulate urination and pain perception from the urinary bladder. On the other hand, the efferent functions include local regulation of the activity of muscle cells, excitability of nerve blood flow and leakage of plasma proteins [106]. Initial characterization of TRPV1 receptors in the urinary tract was performed by radioligand binding assays using [3H]-RTX in membranes from urinary bladder of rats [107]. Subsequently, immunohistochemistry and electron microscopy studies revealed labeling of nerve fibers innervating the bladder muscles and basal urothelial cells [108,109]. These fibers can be located inside or around the bladder mucosa in proximity to blood vessels and smooth muscle cells [108]. TRPV1 is also expressed in non-neuronal cells of the bladder, such as urothelial cells and myofibroblasts [109,110,111,112,113,114]. TRPV1 immunoreactivity was also found in a population of cells located in suburothelial space which possess characteristics of myofibroblasts and are electrically active. These cells could serve as an electrical network capable of modulating bladder sensations [115,116]. However, recently the cellular localization of TRPV1 expression in the bladder has been debated. Different studies using patch-clamp, Ca^2+^ imaging, RT-PCR, immunohistochemistry and Western blotting found no evidence for the functional expression of TRPV1 in bladder urothelium. Further studies are warranted to support these findings and clarify the role of TRPV1 in normal bladder physiology as well as pathologies [114,117,118].

Cultures of urothelial cells from rats and mice exhibit increased intracellular Ca^2+^ and nitric oxide production when challenged with selective TRPV1 agonists. No responses were observed in urothelial cells obtained from TRPV1 knockout animals [109], implicating TRPV1 in this process. Bladder distention leads to greater activity of afferent nerves which was attenuated by capsazepine and was abolished in TRPV1 knockout animals [119]. In animal models, capsaicin induces a transient increase in the frequency of bladder contractions and reduces the volume threshold needed to induce voiding [120,121,122].

The role of TRPV1 receptors has also been analyzed in various urinary tract pathologies. Patients with neurogenic detrusor overactivity exhibit increased expression of TRPV1 in urothelial cells and nerve fibers, compared to healthy subjects. Administration of RTX to these patients reduced the levels of TRPV1 in these areas [110]. Rats with detrusor muscle overactivity exhibited reduced amplitudes of bladder contraction when treated with the TRPV1 antagonist GRC-6211 [123]. Interestingly, individuals suffering from overactive bladder were effectively treated with TRPV1 agonists, capsaicin or RTX [124], which might have the capacity to destroy TRPV1 expressing neurons. Cultures of urothelial cells from patients with non-congenital bladder overactivity exhibit greater expression of TRPV1 compared with the control group and increased sensitivity to capsaicin [125].

Cystitis is an inflammation of the bladder which produces visceral pain. Cystitis induced by cyclophosphamide (a chemotherapeutic agent) in mice and rats is characterized by mechanical bladder hyperactivity which is antagonized by pretreatment with selective TRPV1 antagonist SB-366791, and is not observed in TRPV1 null mice. In contrast, the TRPV1 null mice did not show any deficits in their development of bladder inflammation [126,127]. The induced cystitis was associated with increased TRPV1 channel activity in neurons innervating the bladder and the associated dorsal root ganglia [128], along with increased TRPV1 expression in the bladder [129]. Overall, these data indicate that TRPV1 plays a principal role in the development of pain related to cystitis, suggesting the utility of TRPV1 blockers to treat this condition.

### 3.5. TRPV1 in the Lung

Nerve fibers expressing TRPV1 innervate different components of the respiratory tract, including the trachea, bronchi, alveoli, smooth muscles and blood vessels [130]. These receptors are also expressed in lung and cells lining the airway, but at a relatively lower level [131,132]. Interestingly, patients with emphysema (and are smokers) have a higher expression of TRPV1 receptors when compared to non-smokers [133]. Polymorphisms in the TRPV1 receptor gene are associated with a higher incidence of coughs in patients without asthma, smokers and individuals exposed to environmental irritants such as vapors, gases and dusts [134]. A recent study demonstrated a functional association between a specific polymorphism, TRPV1-I585V, with childhood asthma. Asthmatic subjects with this polymorphism exhibit a lower risk of coughing and wheezing. This result could be explained by a reduced activity of this polymorphic channel compared to the normal TRPV1. The polymorphism rendered these channels less responsive to activation by heat and capsaicin, indicating that TRPV1 plays an important role in the pathophysiology of asthma [135].

A number of studies relating to TRPV1 in the airway focus on its role on sensory nerves which stimulate the cough reflex. McLeod *et al*. [136] demonstrate that capsaicin can induce cough in guinea pigs by activating TRPV1. Capsaicin and citric acid were also capable of inducing cough, which was blocked in a dose-dependent manner by pretreatment with the antagonist iodo-resiniferatoxin (I-RTX) [137]. Similar effects were produced by JNJ-17203212 and capsazepine, two other TRPV1 selective antagonists [138,139]. Furthermore, in an *in vivo* asthma model, TRPV1 antagonist, *N*-(4-tertiarybutylphenyl)-4-(3-chloropyridin-2-yl)tetrahydropyrazine-1(2H)-carbox-amide (BCTC), attenuated the coughing induced by allergens [136]. Increased sensitivity to TRPV1 agonists was observed in airway diseases. For example, patients with upper respiratory tract infection, asthma or chronic obstructive pulmonary disease have increased incidence of cough in response to capsaicin [140,141,142]. Children with allergic rhinitis or upper respiratory infection show worsened coughs stimulated by capsaicin, compared to unaffected children [143,144]. Patients with chronic coughs have increased expression of TRPV1 in nerve fibers [145] and airway muscle cells [146], implicating these receptors in the increased incidence of coughing. The findings in humans were reproduced in animal models of respiratory diseases. For example, exposure of these animals to allergens, tobacco smoke or viral infection causes hypersensitivity to TRPV1 agonists and morphological changes in airway nerves [147,148,149,150,151,152].

## 4. TRPV1 Antagonists

Two methods aimed at blocking TRPV1 in diseases have been pursued as potential treatment options. One method involves activation and desensitization of TRPV1 by agonists. Another method involves the use of antagonist drugs for TRPV1 receptor.

A surprising finding is that capsaicin has traditionally been used to reduce pain, primarily by its ability to desensitize TRPV1. Capsaicin (as 0.025%–0.075% cream preparations) is effective for treating pain produced by osteoarthritis and rheumatoid arthritis and peripheral neuropathy [153], such as diabetic peripheral neuropathy [154]. Topical capsaicin preparations have also been shown to provide relief of post-herpetic pain [155]. However, burning sensations or erythema at the site of application could decrease wide spread application of this latter treatment option [156].

**Figure 2:**
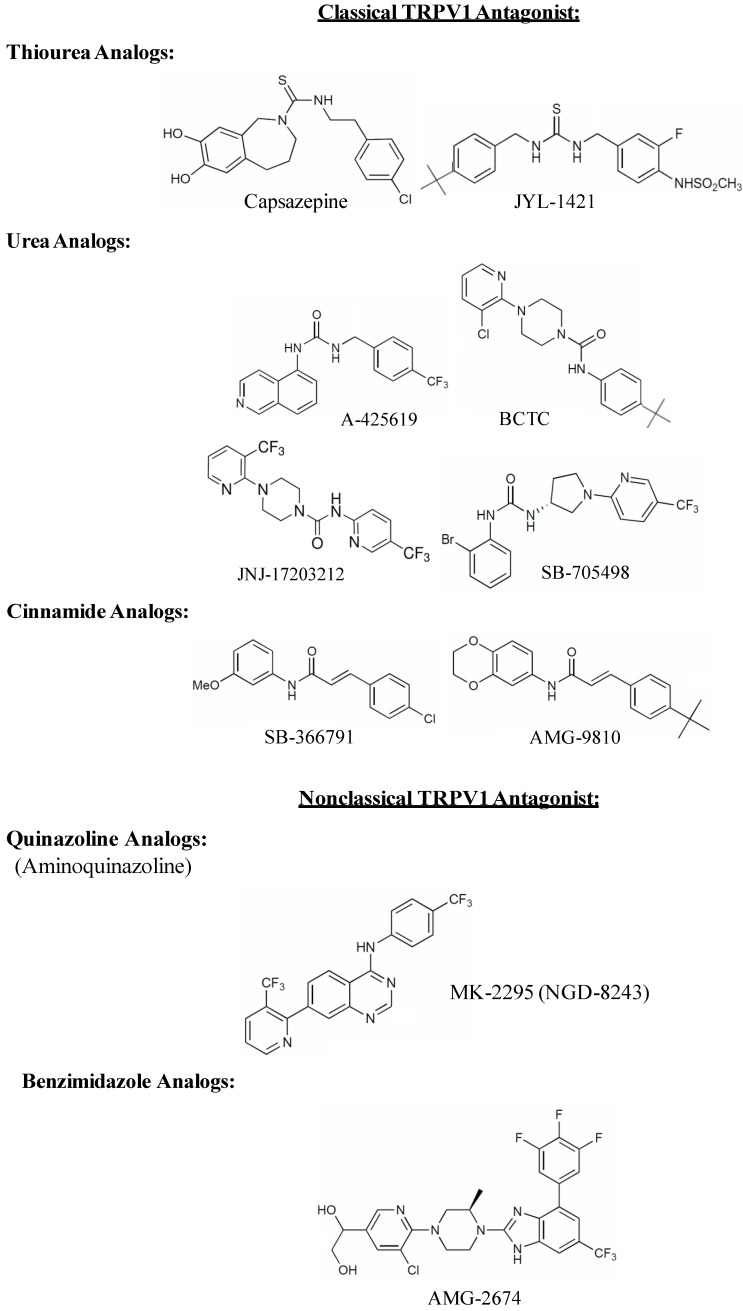
Chemical structures of competitive TRPV1 receptor antagonists.

Pharmaceutical companies have invested millions of dollars for drug screening and lead optimization programs that have identified selective and potent small molecule TRPV1 antagonists, many of which are undergoing clinical trials as analgesic drugs (for review see [157,158,159]). Early structure-activity relationship (SAR) studies to identify TRPV1 antagonists focused on the structure of capsaicin, a naturally occurring TRPV1 agonist. The first reported TRPV1 antagonist, capsazepine, was discovered by modifying the chemical backbone of capsaicin [160]. In capsazepine, the amide bond of capsaicin is replaced by a thiourea moiety, the amide nitrogen of which acts as tether, forcing the aromatic ring to form an orthogonal orientation with respect to the thiourea bond (see Figure 2). This tether was believed to be critical for the antagonistic activity of capsazepine. Capsazepine competes for the capsaicin-binding site on TRPV1, blocks capsaicin-induced channel activation in neonatal rat dorsal root ganglion [161] and displaces RTX from its binding site in radioligand binding studies [34]. Although capsazepine was found to be extremely useful in laboratory research, it was not considered an important candidate for clinical use. One of the reasons is its low metabolic stability and poor pharmacokinetic properties as demonstrated in rodents [23]. Another factor that impeded the clinical use of capsazepine as TRPV1 antagonist was its apparent non-selectivity [157,158,159]. In addition to inhibiting TRPV1, capsazepine also inhibited nicotinic acetylcholine receptors [162], voltage-gated Ca^2+^ channels [163] and TRPM8 [164]. Moreover, capsazepine illustrated species-dependent effects in various models of chronic inflammatory and neuropathic pain [165] possibly due to the species-related differences in the binding of capsazepine to TRPV1. The anti-hyperalgesic effect of capsazepine was more effective in reversing the persistent inflammatory and neuropathic pain in guinea pig than in mice or rats [165]. These shortcomings of capsazepine led to the development of potent and more selective TRPV1 antagonists. The tether, which was believed to be critical for the antagonistic effect of capsazepine, was later found to be irrelevant, as a number of compounds lacking this feature emerged as better antagonists than capsazepine, possessing excellent therapeutic potential in pain regulation and considered as promising clinical candidates [166,167].

Despite its drawbacks, the SAR of capsazepine was used as a template for developing next generation TRPV1 antagonists. A pharmacophore model for the structure of an ideal TRPV1 antagonist has been proposed based on its key binding interactions with the ion channel. This model is combined with the homology model of the TRPV1 channel which is used to filter the set of possible antagonists both by size and shape of the site and by location of appropriate interacting sites on the protein [168]. According to this model, a unifying structural feature of TRPV1 antagonists emerged that has a central hydrogen-bond acceptor/donor motif flanked by a lipophilic side chain on one side and an aromatic group that incorporates a hydrogen-bond acceptor on the other side [23,169].

TRPV1 antagonists can be broadly classified as competitive or non-competitive antagonists. A competitive antagonist binds to the agonist binding site and locks the TRPV1 channel in closed, non-conductive state. All major competitive TRPV1 antagonists discovered so far can be divided into two major classes, classical and non-classical antagonists [23]. The classical antagonists are characterized by the presence of a carbonyl group that can act as H-bonding donor or acceptor and which is present in the form of thiourea, urea, ester or amide (see Figure 2). Many early generation TRPV1 antagonists including capsazepine had either thiourea or urea as a key moiety which was believed to be important for activity. These include JYL-1421 [170], A-425619 [171], BCTC [172], JNJ-17203212 [173] and SB-705498 [174]. Another common functionality of classical TRPV1 antagonists is cinnamide (Figure 2), the structural feature of which can be traced back to the prototypical TRPV1 antagonist capsazepine (e.g., SB-366791 [175] and AMG-9810 [176]). Non-classical TRPV1 antagonists on the other hand have a carbonyl group which is either present as a part of heterocyclic ring or is unrecognizable. These are represented as quinazoline [177] or benzimidazole analogues (AMG-2674) [178] (Figure 2), which are structurally different from capsazepine but still retain the key binding elements.

**Figure 3:**
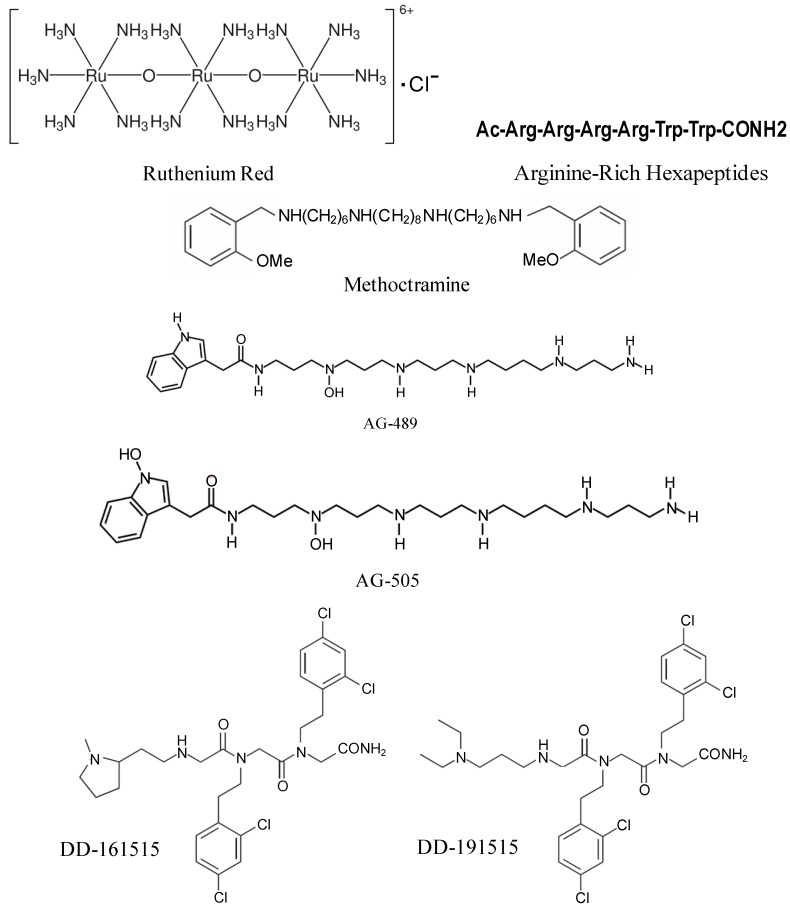
Chemical structures of non-competitive TRPV1 receptor antagonists.

Non-competitive antagonists of TRPV1 channel are pore blockers that interact with additional binding sites (allosteric sites) thereby preventing channel opening by the agonist or blocking its aqueous pore (hence pore blockers). Non-competitive antagonists acting as open-channel blockers are therapeutically more attractive because they preferentially recognize the population of pathologically-over-activated TRPV1 channels and block them, thereby reducing the potential unwanted side effects [179]. The first ever non-competitive TRPV1 antagonist used was a trinuclear polyamine complex, ruthenium red (Figure 3). It is a nonspecific inhibitor of TRP channels that binds to the pore of the channel with high potency in a voltage dependent manner (*i.e.*, it blocks inward currents but not outward currents) [180]. The poor channel selectivity of ruthenium red is thought to be responsible for its pro-convulsive activity in animal models that deterred its clinical development as an analgesic agent [179]. Arginine-rich hexapeptides like RRRRWW-NH_2_ (Figure 3) were identified to block recombinant TRPV1 channels expressed in *Xenopus* oocytes in a non-competitive nonselective manner with submicromolar potency [181]. However, owing to its nonselective nature, like ruthenium red, it showed severe side effects. A parallel approach identified methoctramine (Figure 3), a polymethylene tetraamine, as non-competitive capsaicin antagonist. Methoctramine, a muscarinic M_2_ receptor blocker, antagonized native TRPV1 receptors in DRG neurons activated by either capsaicin or protons in a voltage-dependent manner [182]. The lack of receptor selectivity has restrained its use clinically.

Two acylpolyamine toxins, AG-489 and AG-505 (Figure 3), purified from the venom of North American funnel web spider, *Agelenopsis aperta*, showed robust TRPV1 inhibitory activity by blocking the channel from the extracellular side of the membrane. TRPV1 blockade by AG489 was found to be strongly voltage-dependent, with relief from inhibition being observed at positive voltages. This observation is consistent with a model in which toxin inhibits the channel through a pore-blocking mechanism [23,183].

Screening of a library of trimers of *N*-alkylglycines (also known as peptoids) identified two compounds, DD-161515 and DD-191515 (Figure 3), which preferentially inhibit TRPV1 channel activity with micromolar potency and moderate voltage-dependency [184]. These compounds were effective in treating inflammatory pain induced by injection of capsaicin into the hind paws of mice and also reduced thermal hyperalgesia due to mustard oil-evoked tissue irritation. However, they did not treat capsaicin triggered-mechanical hypersensitivity [184] and the *in vivo* doses required for analgesic and anti-inflammatory effect was too high (≥25 mg/kg) to successfully use these drugs clinically.

There are two most desirable characteristics that a TRPV1 antagonist should have [159]. First, the TRPV1 antagonist should block all modes of channel activation. For example, capsazepine was ineffective in reversing inflammation-based pain behavior in rats [165,185]. In addition, capsazepine demonstrates species difference in its ability to block multiple modes of TRPV1 activation (discussed above). In contrast, other chemically distinct compounds, such as A-425619, AMG-9810 and BCTC, not only inhibited all modes of rat TRPV1 activation but also inhibited and reduced inflammation-related hyperalgesia in rats [176,186,187]. Thus, in order to achieve significant analgesic effect, the TRPV1 antagonists that inhibit all modes of TRPV1 activation are more desirable. 

Another critical feature that is desirable in TRPV1 antagonists is its brain-penetration. The antagonists that penetrate the brain shows more analgesic efficacy than the ones whose actions are limited to the periphery. For example, the TRPV1 antagonist A-784168 showed good CNS penetration and hence was reported to be more potent in inhibiting pain that was presumably mediated by central sensitization than A-795614, a peripherally restricted TRPV1 antagonist [188]. However, both the compounds were equally potent when administered intrathecally, suggesting that brain penetration provides better efficacy.

In addition to the transmission of pain, TRPV1 plays an independent role in regulating body temperature. It is well known that TRPV1 agonist, capsaicin, transiently decreases body temperature in different species, including man [189]. While studying the ability of TRPV1 antagonists to inhibit capsaicin-induced hypothermia, it was observed that some of them caused hyperthermia [190,191]. These findings were surprising due to the fact that TRPV1 knock-out mice showed no difference in core body temperature than the wild-type mice [36]. Interestingly, TRPV1 antagonist, AMG-0347 and AMG-517 did not induce hyperthermia in TRPV1 knock-out mice, suggesting that the TRPV1 antagonist-mediated hyperthermia is TRPV1-dependent [192]. The hyperthermia caused by highly potent and TRPV1-selective antagonists AMG-0347 and AMG-517 were reportedly due to vasoconstriction (results in decreased heat loss through skin) and increased thermogenesis (increased metabolic heat production). Another drawback of using TRPV1 antagonists is that it elevates the threshold for detection of noxious heat [193], which could raise the possible complication of accidental burn injuries in susceptible patients. This elevation of heat threshold was reported to be pronounced for some TRPV1 antagonists than for others [194]. The question whether the hyperthermic action of TRPV1 antagonists can be separated from their analgesic action is still unanswered. Meanwhile, various strategies have been tested to alleviate the hyperthermia caused by TRPV1 antagonists while still preserving their analgesic properties. TRPV1 antagonist-induced hyperthermia is responsive to anti-pyretic agents like acetaminophen [190]. Hyperthermia caused by TRPV1 antagonists desensitizes after repeated administration of antagonists [190]. Another attractive approach is to chemically modify the pharmacophore structure of TRPV1 antagonists in order to prevent the undesirable side effect of hypothermia while inhibiting all modes of TRPV1 activation.

## 5. Conclusions

Since its cloning over a decade ago, research on TRPV1 has grown considerably. While a primary area of interest is the role of this channel in mediating pain, especially inflammatory and chronic pain, a number of researchers are studying the ability of agonist or antagonists of these receptors to relieve symptoms of diseases ranging from diabetes and urinary incontinence to arthritis and hearing loss. The rapid growth of research in these areas bodes well of the development of effective TRPV1 drugs to treat these and other diseases. However, the full adoption of TRPV1 antagonists into clinical practice would depend on the development of effective measures to counter drug-induced hyperthermia.
